# Ratiometric optical probes for biosensing

**DOI:** 10.7150/thno.82323

**Published:** 2023-04-29

**Authors:** Xiao Yang, Congcong Li, Peifeng Li, Qinrui Fu

**Affiliations:** Institute for Translational Medicine, The Affiliated Hospital of Qingdao University, College of Medicine, Qingdao University, Qingdao 266021, China.

**Keywords:** optical probes, ratiometric probes, biosensing, self-calibration, analytes

## Abstract

Biosensing by optical probes is bringing about a revolution in our understanding of physiological and pathological states. Conventional optical probes for biosensing are prone to inaccurate detection results due to various analyte-independent factors that can lead to fluctuations in the absolute signal intensity. Ratiometric optical probes provide built-in self-calibration signal correction for more sensitive and reliable detection. Probes specifically developed for ratiometric optical detection have been shown to significantly improve the sensitivity and accuracy of biosensing. In this review, we focus on the advancements and sensing mechanism of ratiometric optical probes including photoacoustic (PA) probes, fluorescence (FL) probes, bioluminescence (BL) probes, chemiluminescence (CL) probes and afterglow probes. The versatile design strategies of these ratiometric optical probes are discussed along with a broad range of applications for biosensing such as sensing of pH, enzymes, reactive oxygen species (ROS), reactive nitrogen species (RNS), glutathione (GSH), metal ions, gas molecules and hypoxia factors, as well as the fluorescence resonance energy transfer (FRET)-based ratiometric probes for immunoassay biosensing. Finally, challenges and perspectives are discussed.

## Introduction

Sensing based on disease-associated analytes is an effective method for early diagnosis and subsequent treatment decision 1. Biosensing has been one of the hot research topics in disease diagnostics, detection, and other biomedical fields, as a technique that generates signals associated with specific biomolecules of a disease and enables disease diagnosis by identifying the presence or change in concentration of disease-related analytes 2, 3. Highly sensitive biosensing of clinically relevant analyte concentration changes is essential to ensure accurate disease diagnosis and reliable health monitoring 4, 5. With the development of materials science and imaging technology, optical modalities such as fluorescence (FL) probes and chemiluminescence (CL) probes have been successfully integrated into biosensing 6-8. Biosensing based on optical probes is bringing about a revolution in our understanding of physiological and pathological states.

In the last decade, optical probes have received widespread attention due to their advantages such as high sensitivity, high spatial and temporal resolution, and their wide range of applications in qualitative and quantitative sensing of disease-related analytes 9-12. Optical probes can be divided into photoacoustic (PA) probes 13, photoluminescent (FL) probes 14, and self-luminescent probes 15 including bioluminescence (BL) probes, CL probes and afterglow probes, according to different imaging modalities. ​Optical probes with a single sensing signal may cause inaccurate assay results, which are susceptible to target-independent factors such as excitation source fluctuations, the specific microenvironment surrounding the probes, and variations in the local concentration of the probes 16, 17. Ratiometric optical probes have become an important tool for biosensing due to their excellent built-in self-calibration signal correction capability to overcome the limitations of optical probes with a single sensing signal 18, 19. Ratiometric optical probes are based on analyte content changes in signal intensity caused by the acquisition of targets in two or more emission/absorption bands at different wavelengths, thus the influence of various external factors on the sensing results is avoided and the sensing accuracy is significantly improved 20-23. Considering the many advantages of ratiometric optical probes, they can be used as a promising tool for fundamental applications and clinical research in biomedical fields.

**Figure 1:**
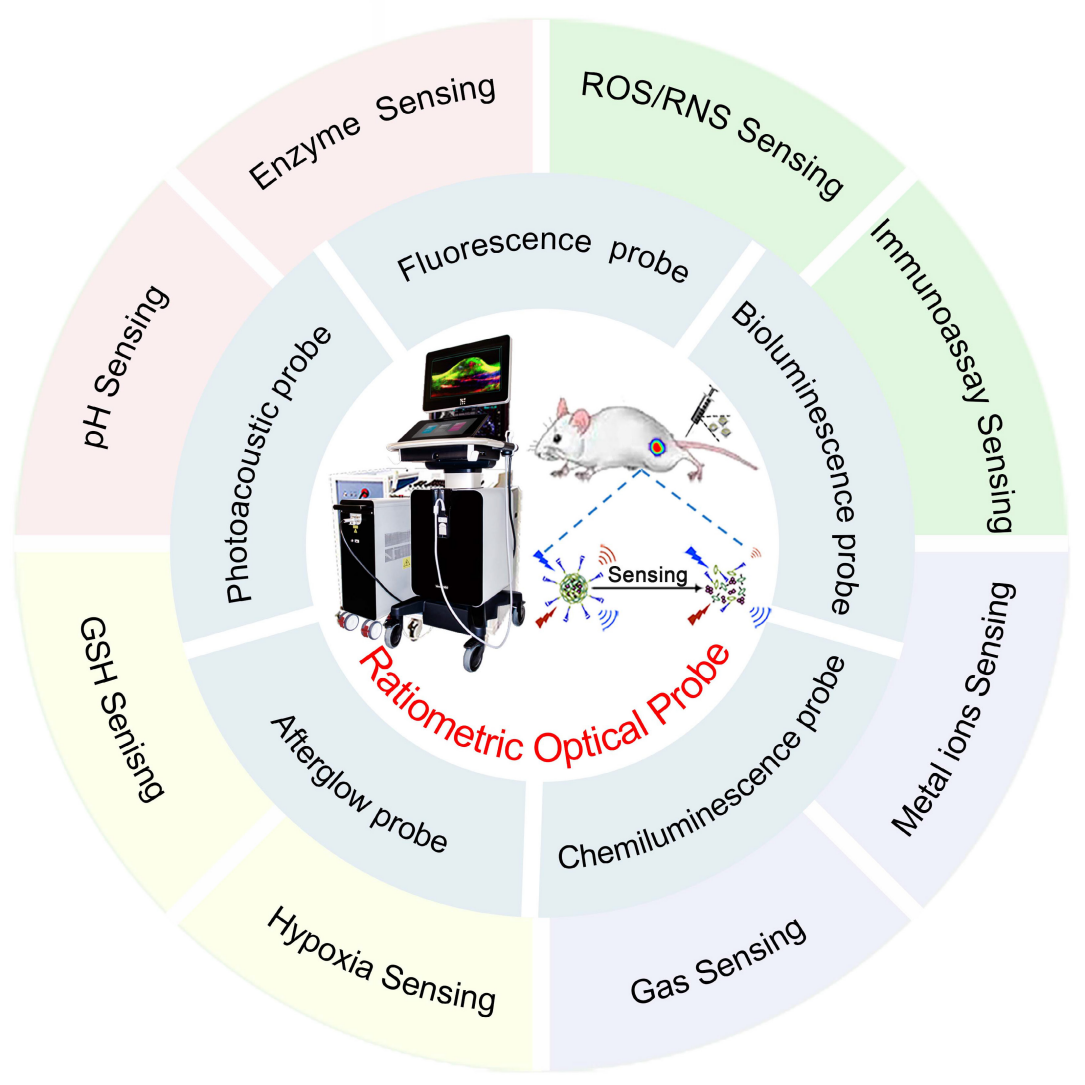
Schematic illustration of ratiometric optical probes (PA probes, FL probes, BL probes, CL probes and afterglow probes) for biosensing such as sensing of pH, enzymes, ROS, RNS, GSH, metal ions, gas molecules, hypoxia factors and immunoassay.

Although there have been numerous reports on ratiometric probes in the recent years, most of which focus on specific types of probes or specific applications, to the best of our knowledge, few reviews have comprehensively summarized ratiometric optical probes for biosensing. Herein, we focus on the design principles of recently reported ratiometric optical probes and their applications in biosensing. The advancements and sensing mechanism of optical probes (Table 1) including PA probes, FL probes, BL probes, CL probes and afterglow probes are first discussed. The versatile design strategies of these ratiometric optical probes are discussed along with a broad range of applications for biosensing such as sensing of pH, enzymes, reactive oxygen species (ROS), reactive nitrogen species (RNS), glutathione (GSH), metal ions, gas molecules, hypoxia factors and immunoassay (Figure 1). Finally, challenges and perspectives are discussed.

### Classification of ratiometric optical probe

Ratiometric molecular probes and ratiometric nanoprobes are collectively referred to as ratiometric probes. Molecular probes are systems that form strong and inherently irreversible bonds with their target analyte 40; Nanoprobes are constructed primarily by functionalizing nanomaterials with specific molecular ligands 41, 42; Alternatively, some nanoprobes can be obtained by combining molecular probes with nanoparticles 43, 44. Therefore, in the following sections, the molecular probes and the nanoprobes are represented as probes. Ratiometric optical probes are classified according to different mechanisms as ratiometric PA probes, ratiometric FL probes, and ratiometric self-luminescent probes including ratiometric BL probes, ratiometric CL probes and ratiometric afterglow probes.

### Ratiometric PA probes

**Figure 2:**
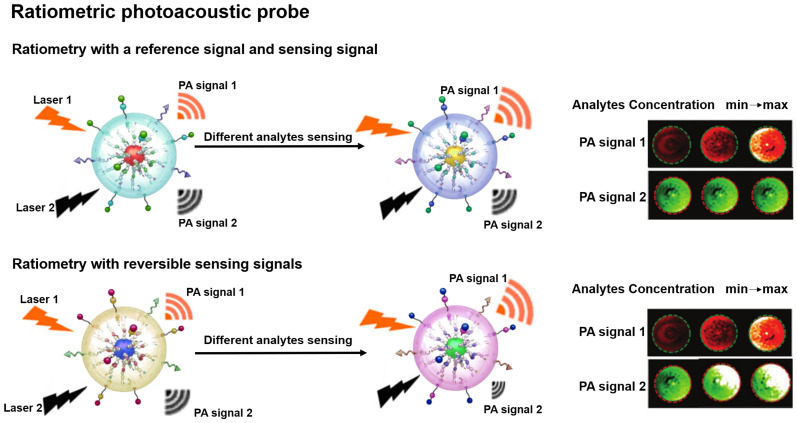
Schematic illustration of ratiometric PA probes for sensing *in vitro* and *in vivo* and the PA signal changes with the concentration of analytes. Adapted with permission from 68, Copyright 2019 American Chemical Society.

PA probe is a non-invasive biomedical diagnostic tool that utilizes the PA effect to convert absorbed photons into sound waves 45, 46. ​​The PA probe combines the advantages of optical and ultrasound with the features of strong contrast and high spatial resolution to provide a favorable strategy for the study of physiological and pathological states of organisms 47-50. ​Ratiometric PA probe is based on self-calibration of the signal intensity and recording of the signal fluctuations induced by the analytes 51. There are two design strategies for the ratiometric PA probe: one strategy is that a single probe with an analyte-insensitive reference signal and an analyte-responsive sensing signal to achieve the ratio measurement 52; another strategy is that the probe achieves the ratio measurement by reversible signal changes in the responses of an analyte 53. The built-in self-calibration of ratiometric PA probes enables more sensitive and reliable detection, providing an essential means to study the morphological structure, metabolic function, physiological and pathological properties of biological tissues (**Figure 2**) 54-56.

### Ratiometric FL probe

**Figure 3:**
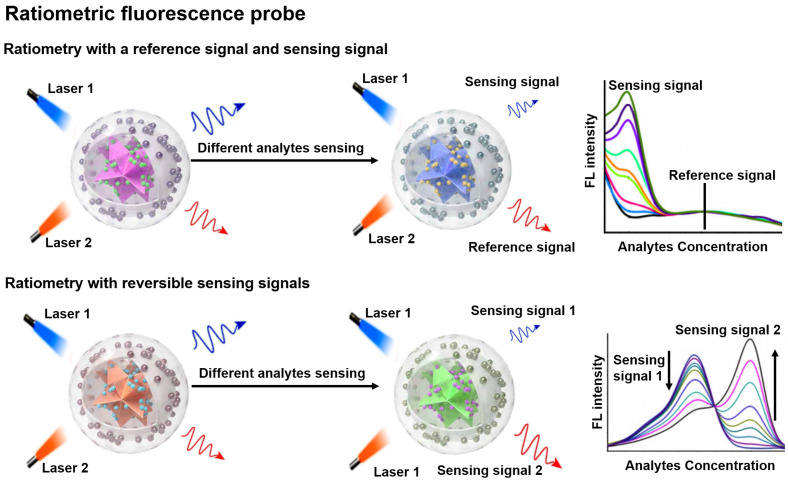
Schematic representation of the sensing principle of ratiometric FL probes and the FL intensity varies with the analyte concentration. Adapted with permission from 69, Copyright 2022 Wiley-VCH.

FL is a photoluminescent phenomenon that relies on the emission of excited singlet relaxed photons 57, 58. Compared to non-optical probes, FL probes offer superior sensitivity and strong spatial resolution, enabling non-invasive and real-time sensing of biological tissues 59-62. ​Traditional FL probes are susceptible to analytically independent factors that cause drastic changes in the intensity of the background signal, which in turn affect the FL signal results. Ratiometric FL probes can adequately overcome the above issues by combining the reference and sensing signals, or by incorporating both reversible signal variations in response to the analytes into a single probe (**Figure 3**) 18, 63, 64. ​The proposed mechanism of ratiometric FL probes can effectively improve the sensing contrast and provide high detection sensitivity for disease diagnosis, and will have a large scope in biomedical applications 65-67.

**Figure 4:**
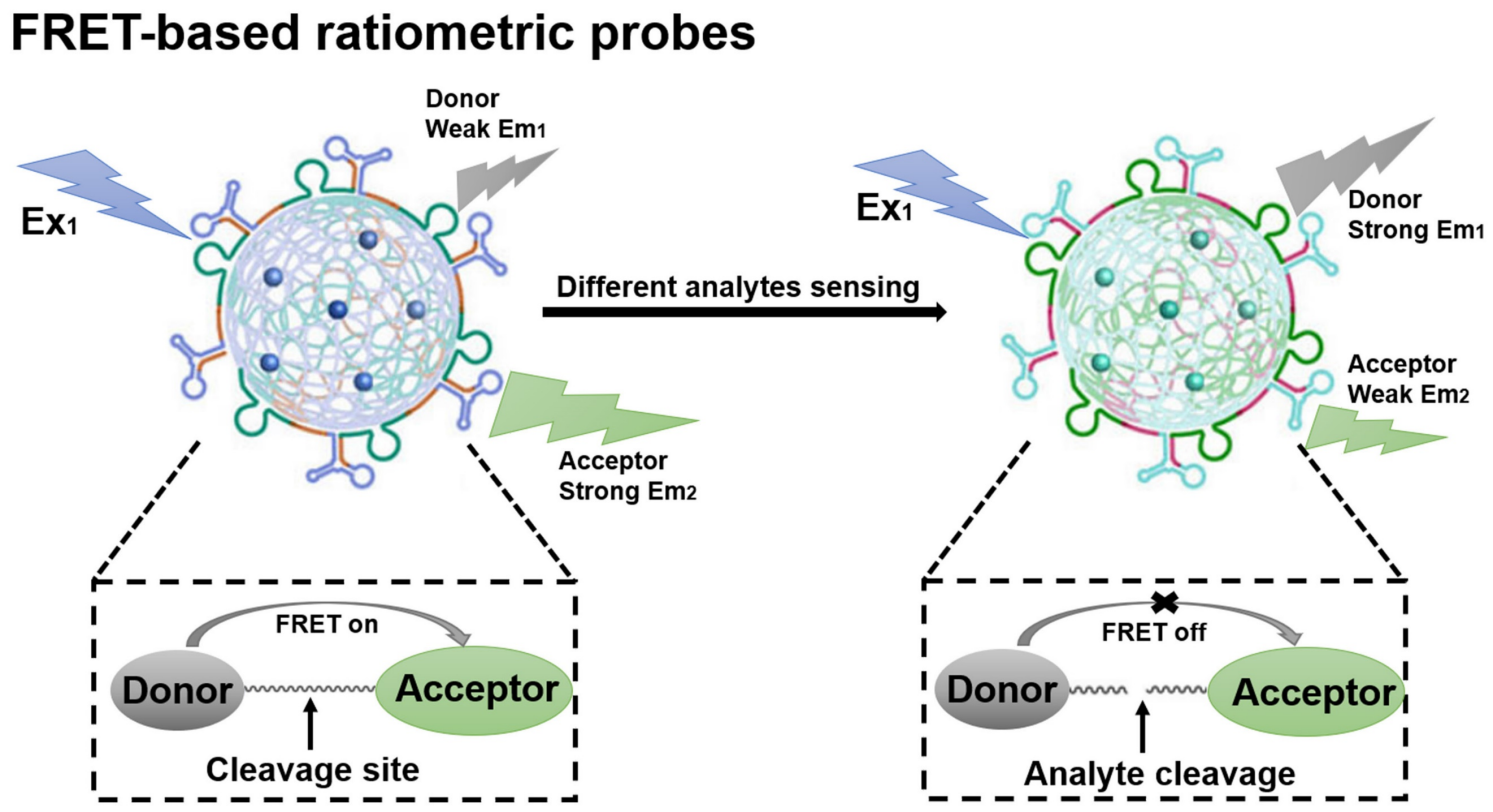
Schematic illustration of the sensing principle of FRET-based ratiometric probes. The cleavage of the covalent link between the donor and acceptor by the analyte causes the FRET system to shut down. Adapted with permission from 73, Copyright 2023 American Chemical Society.

Fluorescence resonance energy transfer (FRET)-based probes belong to the class of FL probes, which are small molecule probes designed based on the FRET effect and have been widely used for selective detection in living organisms 70. The general principles for the design of FRET-based ratiometric probes are the proximity of the donor-acceptor pair and the overlap of the emission spectrum of the donor with the absorption spectrum of the acceptor, as well as the need for a reaction unit that can be used as a specific recognition group 71. When a FRET probe is exposed to a disease-relevant target analyte, the FRET effect is initiated or blocked and, in turn, a change in the FL signal ratio can be observed, which ultimately determines whether the target analyte is present or not (**Figure 4**) 72. FRET-based ratiometric probes thus provide a unique way to sense biologically and environmentally important analytes and can reveal the physiological and pathological function of these analytes.

### Ratiometric self-luminescence probe

**Figure 5:**
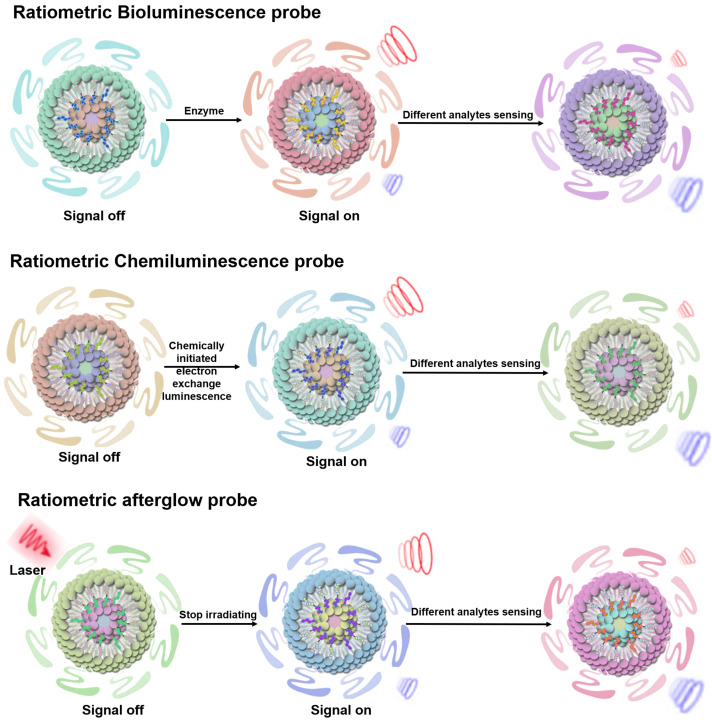
Schematic illustration of the sensing principle of ratiometric self-luminescence probes. Adapted with permission from 84, Copyright 2022 American Chemical Society.

BL probes, CL probes and afterglow probes all belong to the category of self-luminescent probes, which can effectively eliminate external light interference and achieve high sensitivity sensing results 74. BL refers to light emitted by catalytic reactions between bioluminescent enzymes (luciferase) and substrates (luciferin and other small molecules) 75-77. In BL process, the energy generated by an enzymatically catalyzed chemical reaction excites electrons to excited state, and photons are emitted as light when the electrons return to their ground state 78. CL can be described as the phenomenon of emission of photons when electrons return from the excited state to the ground state during a chemical reaction. Compared to photoluminescent probes, CL probes are a valuable sensing tool because they provide their own light source and thus attenuate autofluorescence and light scattering effects 79, 80. ​Afterglow luminescence requires pre-illumination of the afterglow agent. The excitation energy in defects is captured by the afterglow agents, and the stored energy is slowly released by photons during physical activation, thus eliminating autofluorescence 81, 82. ​Ratiometric self-luminescent probes typically integrate analytically sensitive signals as sensing signals and analytically insensitive signals as reference signals into a single probe, or a single probe with analytically responsive reversible signal variations, which do not rely on real-time excitation of exogenous light to detect the luminescence signal, and can effectively eliminate the interference of self-luminescence and achieve high-sensitivity sensing (**Figure 5**) 38, 83.

### Ratiometric optical probes for biosensing

​Abnormal pH, ROS, RNS, GSH, enzymes, metal ions, gases, and hypoxia factors in the tumor microenvironment (TME) plays a major role in managing homeostasis processes 85-87. Thus, the sensing of pathological factors in the TME provides new insights for the diagnosis or discovery of certain diseases. Recently, ratiometric optical probes have been frequently used for the sensing of endogenous pathological factors due to their advantage of being free from external environmental interference, leading to more reliable and accurate sensing results 88, 89.

### pH sensing

**Figure 6:**
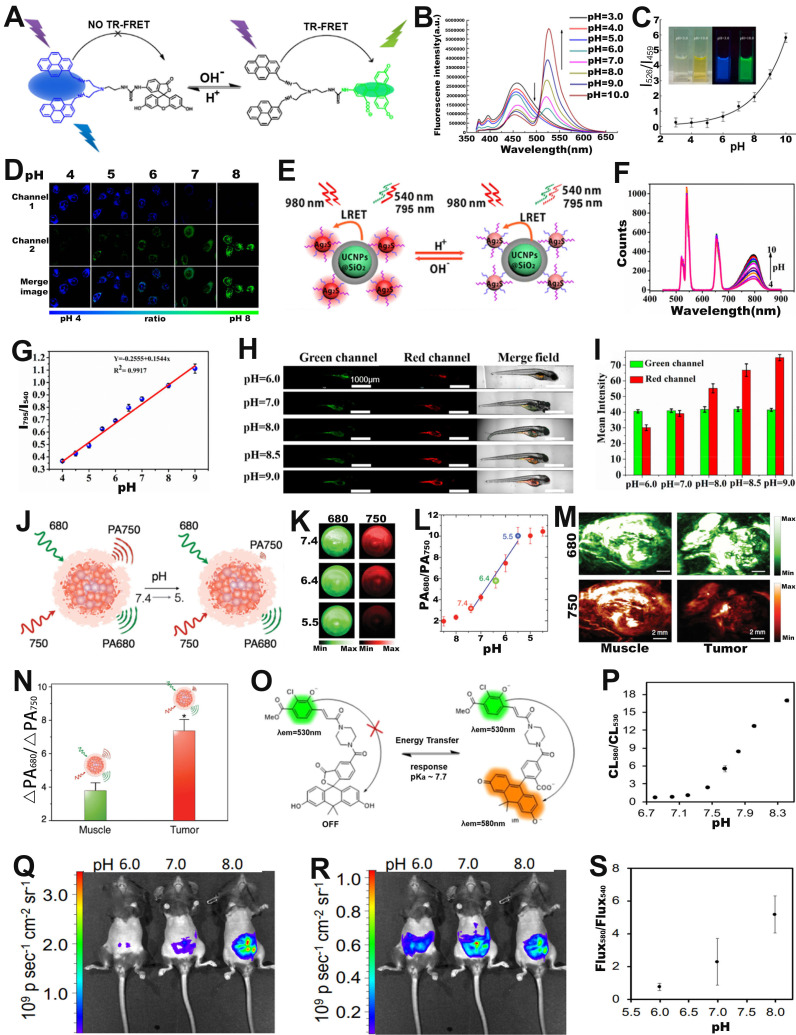
(**A**) The structure and response mechanisms of FRET-based ratiometric bispyrene-fluorescein hybrid probe. (**B**) The FL intensity changes of PF probe with pH value. (**C**) The relationship between pH and FL signal ratio (I_526_/I_459_). (**D**) Confocal FL image after incubation of probe with Hela cells. (**E**) Schematic diagram of pH sensing mechanism of UCNPs@SiO_2_-Ag_2_S probe. (**F**) The luminescence spectrum changes with pH value. (**G**) The FL signal ratio as a function of pH. (**H**) Confocal images of zebrafish with probes incubated at different pH, and (**I**) corresponding luminescence intensity ratio. (**J**) Proposed mechanism of ratiometric probe SNOS for pH sensing. (**K**) PA imaging at different pH values. (**L**) The relationship between pH value and PA signal ratio. (**M**) PA images of muscle and tumor site after injection of probe, and (**N**) corresponding PA_680 nm_/PA_750 nm_ ratio. (**O**) Chemical structure and sensing mechanism of ratiometric CL probe. (**P**) Relationship between pH and CL_580 nm_/CL_530 nm_. CL images of ratio-pHCl-1 injected into the mice using a (**Q**) 580 nm filter or (**R**) 540 nm filter in the IVIS Spectrum. (**S**) The relationship between pH value and Flux_580_/Flux_540_. (**A**-**D**) Adapted with permission from 91, Copyright 2014 American Chemical Society. (**E-I**) Adapted with permission from 97, Copyright 2019 American Chemical Society. (**J-N**) Adapted with permission from 100, Copyright 2016 WILEY-VCH. (**O-S**) Adapted with permission from 101, Copyright 2020 American Chemical Society.

Intracellular pH is closely related to cellular growth, differentiation and proliferation, and FRET-based ratiometric imaging using pH-sensitive probe provides a powerful tool to assess the pH of cancer cells 90. For example, Yu and co-workers successfully prepared a FRET-based ratiometric bispyrene-fluorescein hybrid probe (PF), consisting of a pH-insensitive bispyrene unit as an energy donor and a pH-sensitive fluorescein as an energy acceptor, for sensing of pH changes in HeLa cells (**Figure 6A**) 91.* In vitro* emission spectrum analysis demonstrated that the emission from fluorescein at 526 nm remarkably enhanced with increasing pH, whereas that from pyrene excimer at 459 nm was significantly reduced (**Figure 6B**). This is due to the change in the structure of the fluorescein cyclolactam in the PF probe from the ring closure to ring-opening with increasing pH, which turns on the FRET effect, resulting in a decrease in the blue emission intensity of the pyrene molecule as a donor and an increase in the green emission intensity of the fluorescein as an acceptor. As shown in **Figure 6C**, the corresponding FL signal ratio (I_526_/I_459_) thus increased from 0.26 to 5.82 as the pH increased from 3 to 10. In addition, the potential of the intracellular ratiometric FL signal for pH sensing was further evaluated. ​As shown in Figure 6D, as the pH increased from 4.0 to 8.0, the signal strength within the HeLa cell from the green channel increased significantly, whereas the that from the blue channel decreased significantly, resulting in a significant enhancement of the ratio from 0.27 to 2.25. ​These results demonstrated the potential of PF probes for quantitative sensing of pH changes in living cells with a wide range of applications in the biomedical field.

FRET-based ratiometric probes can not only sense pH changes at the cellular level, but can also be applied at the *in vivo* level to provide important information on the occurrence and development of diseases 92-94. Among them, upconversion nanoparticles (UCNPs) with excellent capability of bioanalysis and bioluminescence have drawn increasing attention 95, 96. For example, Xian and co-workers fabricated a FRET system (UCNPs@SiO_2_-Ag_2_S) consisting of GSH and mercaptopropionic acid (MPA) co-modified Ag_2_S nanodots (GM-Ag_2_S NDs) as energy acceptor and silica-encapsulated UCNPs as energy donor for real-time sensing of pH in tumor-bearing zebrafish (**Figure 6E**) 97. Under excitation at 980 nm, the GM-Ag_2_S NDs, as a sensing signal, showed an enhanced luminescence intensity at 795 nm with increasing pH; while the UCNPs, as a reference signal, showed a constant signal at 540 nm (**Figure 6F**). As a result, its luminescence signal ratio (I_795_/I_540_) increased as a function of pH (**Figure 6G**). After injecting the probes into zebrafish, the changes in pH *in vivo* were observed *via* ratiometric FL imaging. As shown in **Figure 6H, I**, the green signal at 540 nm remained almost unchanged, while the red signal at 795 nm increased with the increasing pH, resulting in a pH-dependent enhanced I_795_/I_540_ ratio. The results demonstrated that FRET-based ratiometric probes can be used for real-time *in vivo* pH sensing and significantly advance the application of ratiometric optical probes in biomedicine.

​Energy acceptors and donors for FRET-based probes can be provided not only by fluorescent molecules or nanoparticles, but also by fluorescent proteins 98. Fluorescent proteins are extremely well suited for use as optical sensors in living cells due to their unique optical signaling mechanism and their vulnerability to the surrounding chemical environment and protein-protein interactions upon expression. For example, Malli *et al*. designed a ratiometric fluorescent protein FRET biosensor (pH-Lemon) consisting of a pH-insensitive cyan fluorescent protein (CFP) variant (mTurquoise2) and a highly pH-sensitive enhanced yellow fluorescent protein (EYFP) for sensing the local pH dynamics of subcellular microstructures in living cells 99. At neutral to alkaline pH values, the C-terminus of the mTurquoise2 (FRET donor) was fused to the N-terminal end of the EYFP (FRET acceptor) via a small, flexible linker, leading to yield high FRET effect. Under acidic conditions, the pH-sensitive EYFP underwent protonation, which significantly reduced yellow FL, while cyan FL increased due to FRET effect was quenched; the corresponding FL signal ratio (F_EYFP_/F_CFP_) thus decreased with decreasing pH values. These results indicated that fluorescent protein-based ratiometric FL probes could be used for sensing of pH changes.

​ In addition to ratiometric FL probes, ratiometric PA probes can also be used for pH sensing *in vivo*. For example, Pu *et al.* developed an activatable PA probes (SNOS) consisting of an inert PA-based semiconducting oligomer (SO), an amphiphilic triblock copolymer (PEG-b-PPG-b-PEG), and a pH indicator and enhancer (pH-BDP) for *in vivo* sensing of pH changes (**Figure 6J**) 100. As shown in **Figure 6K**, the PA signal at 680 nm remained almost unchanged, while the signal at 750 nm decreased significantly with decreasing pH, due to the fact that the pH-BDP underwent an efficient protonation-deprotonation process with decreasing pH, hence resulting in an increase of the PA_680nm_/PA_750nm_ ratio as a function of pH. As shown in **Figure 6L**, the PA signal ratio at pH = 5.4 was increased by a factor of 3.44 compared to that at pH = 7.4, while the PA signal ratio at pH = 5.5 was increased by 1.91-fold compared to that at pH = 6.4. Therefore, the PA_680nm_/PA_750nm_ ratio can be used to efficiently sense pH in physiological and pathological conditions. After local injection of the probe into the muscles or into the Hela xenografts tumor of nude mice, the intensity of the PA signal at 750 nm in Hela xenografts tumor was significantly reduced compared to that in the muscle tissue (**Figure 6M**), resulting in the PA_680nm_/PA_750nm_ ratio of tumor was 1.9-fold higher than that of muscle tissue (**Figure 6N**) due to tumor acidic microenvironment. In addition, the PA_680nm_/PA_750nm_ ratio showed an excellent linear relationship with the pH value, thus enabling *in vivo* sensing of pH changes *via* ratiometric PA signals.

In addition to ratiometric photoluminescence (FL) probes and ratiometric PA probes, ratiometric self-luminescence probes can also be used for *in vivo* sensing of pH changes. For example, Lippert and colleagues developed a pH responsive ratiometric CL probe with a chemiluminescence resonance energy transfer (CRET) effect (named as ratio-pHCL-1) consisting of a pH-sensitive carbon fluorescein and a chemiluminescent scaffold (acrylamide 1,2-dioxetane) for pH sensing (**Figure 6O**) 101. The CL intensity at 580 nm increased significantly, while that at 530 nm remained almost unchanged with increasing pH due to the CRET effect, leading to the CL signal ratio (CL_580 nm_/CL_530 nm_) increased as a function of pH (**Figure 6P**). After intravenous administration, the CL imaging at 580 nm (**Figure 6Q**) and 540 nm (**Figure 6R**) were performed by using an IVIS Spectrum. The corresponding ratiometric CL signals (Flux_580 nm_/Flux_540 nm_) in the peritoneal cavity of mice were quantitatively calculated (**Figure 6S**), the results showed that a positive correlation between ratiometric CL signals (Flux_580 nm_/Flux_540 nm_) and *in vivo* pH value. ​This result demonstrated the potential of the ratiometric self-luminescent probes to enjoy a wide range of intracellular and *in vivo* pH sensing applications.

### Sensing of ROS

ROS are endogenously generated essential signaling-reactive molecules, such as hydrogen radicals (·OH), superoxide (O_2_^·-^), hypochlorous acid (HOCl/HClO)/hypochlorite (^-^OCl), singlet state oxygen (^1^O_2_), hydrogen peroxide (H_2_O_2_), *etc.*, which play important roles in regulating numerous events of physiological and pathological processes 102-109. Therefore, the establishment of reliable *in vivo* ROS sensing methods in real-time is of great importance for the diagnosis and treatment of diseases. For example, Zhang and co-workers developed a ratiometric FL probe (ErBG@IR808) consisting of HClO responsive IR808 fluorophores modified Er nanoparticles (ErNPs) with a bioactive Glass (BG) scaffold for ROS sensing 110. There was an absorption competition induced emission (ACIE) effect between IR808 and ErNPs, resulting in quenching of the emission at 1525 nm of ErBG scaffolds excited at 808 nm (F_808ex_). In the presence of HClO, the quenched FL signal of ErBG was recovered under excitation at 808 nm (F_808ex_) due to HClO triggered degradation of IR808, while the 1525 nm emission upon 980 nm excitation (F_980ex_) remained unaffected. Therefore, there was a positive correlation between the FL intensity ratio (F_808ex_/F_980ex_) and the concentration of ClO^-^. After intravenous injection of the probe into the inflammatory mice, F_808ex_ gradually increased with the time after injection and reached the maximum value at 7 h post-injection, while F_980ex_ remained constant, resulting in an increase in F_808ex_/F_980ex_ as a function of time. The results illustrated the excellent capability of the ErBG@IR808 probe to sense HClO secreted by inflammatory cells *via* the ratiometric FL signal.

**Figure 7:**
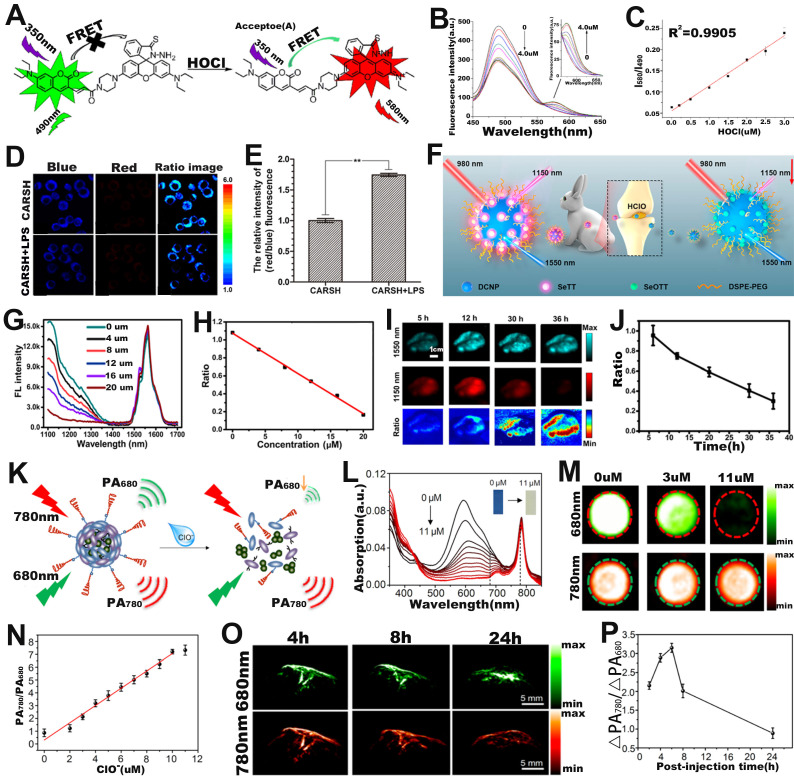
(**A**) Chemical structure and sensing mechanism of CARSH probe. (**B**) The FL intensity changes with the increase of HOCl concentration. (**C**) The relationship between HOCl concentration and FL signal ratio (I_580_/I_490_). (**D**) FL images of RAW264.7 cell after incubation with CARSH probe, and (**E**) corresponding ratio (red/blue) of FL intensity. (**F**) Proposed mechanisms of DCNP@SeTT@PEG probes for sensing of HClO within tumor and inflammation of rabbit models. (**G**) The FL intensity changes with the HClO concentration, and (**H**) corresponding I_1150 nm_/I_1550 nm_. (**I**) FL images in mice after intravenous injection of probe, and (**J**) I_1150 nm_/I_1550 nm_ as a function of time. (**K**) Mechanism of SOA@NIR775 probe for ratiometric PA sensing of ClO^-^. (**L**) Changes in the absorption spectra of probes after the addition of different concentrations of ClO^-^. (**M**) PA images of probes treated with different concentrations of ClO^-^. (**N**) Variation of PA signal ratio with ClO^-^ concentration. (**O**) PA images of subcutaneous 4T1 tumor after intravenous injection of probes, and (**P**) corresponding PA signal ratio. (**A-E**) Adapted with permission from 111, Copyright 2016 Elsevier. (**F-J**) Adapted with permission from 112, Copyright 2020 American Chemical Society. (**K-P**) Adapted with permission from 116, Copyright 2017 American Chemical Society.

FRET-based ratiometric probe can also be used for ROS sensing. For example, Zhao* et al.* developed a ratiometric FL probe (CARSH) consisting of a HOCl-insensitive coumarin-ethylene as FRET energy donor and a HOCl-sensitive rhodamine as FRET energy acceptor for the quantitative sensing of HOCl in living organisms (**Figure 7A**) 111. In the probe, the coumarin-ethylene had an FL peak at 405-550 nm (blue light) and the rhodamine had an FL peak of at 550-650 nm (red light). Upon incubation with HOCl, the thiohydrazide moiety of rhodamine (acceptor) reacted with HOCl to open the thiohydrazide spiro ring, and then the energy of FRET energy donor (coumarin-ethylene) was transferred to the FRET energy acceptor (rhodamine) through the FRET effect, which eventually led to enhanced red-light emission and reduced blue light emission (**Figure 7B**). The FL signal ratio (I_580_/I_490_) increased with the increasing HClO concentration (**Figure 7C**), thus can be used for ROS sensing. RAW264.7 cells were incubated with CARSH probe and CARSH probe + lipopolysaccharide (LPS, which stimulated cells to produce HOCl), respectively, a significant increase in the FL signal ratio (red/blue) corresponding to the group with the addition of LPS was observed (**Figure 7D, E**). The results indicated that the FRET-based ratiometric CARSH probe can be used for sensing of HOCl at the cellular level.

Furthermore, ratiometric FL probe can also be used for sensing of HOCl at the *in vivo* level. Yang and co-workers fabricated a ratiometric FL probe (DCNP@SeTT) consisting of Er^3+^-doped down-conversion nanoparticles (DCNP), polyethylene glycol phospholipids (DSPE-PEG), and a NIR-II FL dye (SeTT) for the effective sensing of ROS *in vivo* (**Figure 7F**) 112. DCNP@SeTT had two specific FL peaks at 1150 nm and 1550 nm, respectively. ​After incubation of the probe with HClO, the FL signal of the SeTT at 1150 nm was weakened due to oxidation of the SeTT by HClO, while the FL signal of the DCNP at 1550 nm, which was used as the internal reference signal, showed negligible changes (**Figure 7G**). Therefore, there was a negative correlation between HClO concentration and FL signal ratio (I_1150 nm_/I_1550 nm_) (**Figure 7H**). After injecting the probe into the mice, the FL images at 1150 and 1550 nm were observed to explore the ability of the probe to sense endogenous HClO (**Figure 7I**). As shown in **Figure 7J**, the presence of overexpressed ROS in the TME resulted in a gradual decrease in the FL signal ratio (I_1150 nm_/I_1550 nm_) with increasing time. These results indicated that the ratiometric FL probe successfully achieved rapid response and high selectivity for HClO sensing under *in vivo* physiological conditions and was a potentially promising tool for diseases diagnosis.

Ratiometric PA probes can also be used for ROS sensing 113-115. For example, Pu and colleagues developed a ratiometric PA probe (SOA@NIR775) consisting of a semiconducting oligomer amphiphile (SOA, with an absorption peak at 680 nm) with a ROS-oxidizable aromatic unit and a ROS-inert dye NIR775 with an absorption peak at 780 nm for *in vivo* ClO^-^ sensing (**Figure 7K**) 116. In the presence of HClO, the ROS-sensitive semiconductor skeleton in SOA was degraded, and the π-π stacking between the semiconductor skeleton in SOA and NIR775 was collapsed. As a result, the PA signal at 680 nm gradually decreased with the increase of HClO concentration, yet the PA signal at 780 nm remained unchanged (**Figure 7L, M**). Therefore, the PA signal ratio (PA_780_/PA_680_) increased linearly with ClO^-^ concentration (**Figure 7N**). As shown in **Figure 7O**, after intravenous administration, the PA signal at 780 nm increased over times, reaching a maximum value at 6 h post-injection, while the signal at 680 nm increased slightly. Therefore, the ratio of PA signal (ΔPA_780_/ΔPA_680_) at 6 h post-injection was 1.47 times higher than that at 2 h post-injection (**Figure 7P**). The results demonstrated that the ratiometric PA probe was an excellent tool to sense ClO^-^ levels under pathological conditions *in vivo* and had promising applications in the process of disease diagnosis.

### Sensing of RNS

**Figure 8:**
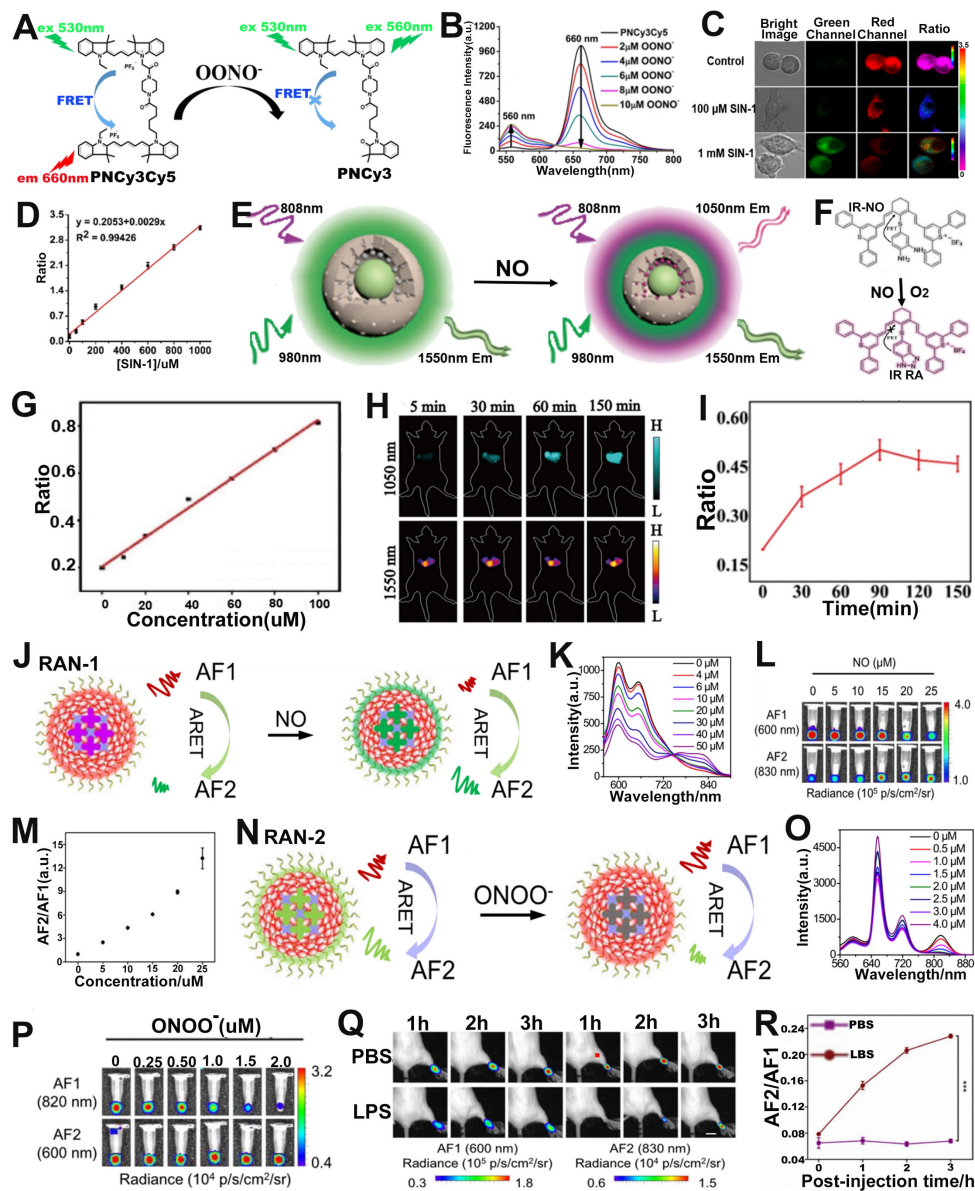
(**A**) Chemical structure and sensing mechanism of PNCy3Cy5 probe. (**B**) FL intensity of PNCy3Cy5 probe after ONOO- activation. (C) Confocal FL imaging of macrophages after different treatments. **(D)** The relationship between SIN-1 concentration and FL signal ratio. (**E**) Schematic illustration of the ratiometric probe for NO sensing. (**F**) Synthetic route and chemical structure of IR-NO. (**G**) Relationship between FL signal ratio and NO concentration. (**H**) Changes in FL images over time after APAP-induced liver injury in mice and (**I**) corresponding FL signal ratio. (**J**) Schematic illustration of ratiometric afterglow RAN-1 probe for NO sensing. (**K**) FL emission spectra of RAN-1 after NO activation. (**L**) Afterglow images of RAN-1 after NO activation. (**M**) Relationship between NO concentration and afterglow signal ratio. (**N**) Schematic illustration of ratiometric afterglow RAN-2 probe for ONOO^-^ sensing. (**O**) FL emission spectra of RAN-2 after ONOO^-^ activation. (**P**) Afterglow images of RAN-2 after ONOO^-^ activation. (**Q**) Changes of afterglow images over time after intravenous administration of RAN-1 in mice pretreated with LPS or PBS, and (**R**) corresponding AF2/AF1. (**A-D**) Adapted with permission from 123, Copyright 2016 American Chemical Society. (**E-I**) Adapted with permission from 124, Copyright 2021 American Chemical Society. (**J-R**) Adapted with permission from 125, Copyright 2022 Springer Nature.

RNS refers to the interaction of nitric oxide (NO) with compounds including ROS, resulting in a series of nitrogen dioxide radicals (NO_2_^•^), peroxynitrite (ONOO^-^), and nitrate (NO_3_^-^) 117-119. ​Overproduction of RNS is closely related to the occurrence of various diseases 120-122, and therefore, there will be a great scope for designing probes with high sensitivity and selectivity for RNS sensing in the biomedical domain. Chang and co-workers synthesized a FRET based ratiometric FL probe (PNCy3Cy5) for ONOO^-^ sensing in living cells (**Figure 8A**) 123. The energy donor (Cy3) and energy acceptor (Cy5) of the PNCy3Cy5 probe was linked by an acetyl piperazinyl hexyl group, and the ratiometric sensing mechanism of PNCy3Cy5 probe was based on modulating FRET between donor and acceptor. Upon incubation with ONOO^-^, Cy5 was selectively oxidized to oxindole derivative and then the FRET effect was quenched, resulting in an increase in the FL emission intensity of Cy3 at 560 nm and a decrease in FL intensity of Cy5 at 660 nm (**Figure 8B**). Therefore, the ONOO^-^ concentration showed a good positive correlation with the FL signal ratio (F_560_/F_660_). In addition, PNCy3Cy5 can specifically sense exogenous OONO^-^ in living cells through ratiometric FL imaging. In an* in vitro* RAW264.7 macrophages experiment, the FL signal ratio (F_560_/F_660_) remarkably enhanced after cells treated with SIN-1 (an OONO^-^ donor) (**Figure 8C**). As shown in **Figure 8D**, a linear relationship between the FL signal ratio (F_560_/F_660_) and the SIN-1 concentration was observed. In addition, the F_560_/F_660_ ratio of untreated and SIN-1 + minocycline (OONO^-^ scavenger) treated RAW264.7 macrophages was lower than that of SIN-1-treated RAW264.7 macrophages. These results demonstrated that PNCy3Cy5 probe could explore the physiological mechanisms of ONOO^-^ and investigate its role in related diseases, indicating its potential for advance biomedical sensing *in vivo*.

The ratiometric FL probe for NO sensing was described by Song *et al*. The probe (DCNP@MPS@IR NO) was comprised of a NO-responsive small-molecule organic dye (IR-NO) as a sensing signal unit, NO insensitive DCNP as a reference signal unit and mesoporous silica (MPS) (**Figure 8E, F**) 124. In the absence of NO, cyanine fluorophore in IR-NO was coupled with the electron donor o-phenylenediamine unit to generate photoinduced electron transfer (PET) effect, resulting in the quenching of FL of cyanine fluorophore. In the presence of NO, the o-phenylenediamine unit in IR-NO can be converted into benzotriazole with weak electron donating capability, resulting in the blocking of the PET process and the restoration of the quenched FL, together with an emission wavelength of 1050 nm at 808 nm excitation. However, DCNP, as an internal reference signal, remained unaffected at 1550 nm upon excitation with a 980 nm laser. Therefore, the ratio signal (F_808ex_/F_980ex_) increased linearly with the increase of NO concentration (**Figure 8G**). Furthermore, *in vivo* liver injury ratiometric FL imaging experiments showed that the FL signal intensity at 1050 nm was enhanced; however, that at 1550 nm remained unchanged (Figure 8H). The FL signal of the probe at 1050 nm displayed a time-dependent increase within 90 min and reached the maximum value at 90 min post-injection. The FL signal ratio (F_808ex_/F_980ex_) thus increased with increasing time post-injection (Figure 8I). These results indicated that the DCNP@MPS@IR NO probe can effectively sense NO in the liver of APAP-induced liver injury mice and can be used for the visualization and early diagnosis of drug-induced liver injury.

Ratiometric afterglow probes are an emerging sensing platform, which can not only solve the problem of attenuation of afterglow intensity, but also eliminate the interference of external factors. Tang *et al.* developed a series of ratiometric afterglow probes (RAN) for NO and ONOO^-^ sensing 125. Among them, RAN-1, consisting of an afterglow substrate (MEHPPV), a NO-responsive molecule (NRM), a surfactant (F127) and an afterglow initiator (AI), can sense NO *in vivo* through the afterglow resonance energy transfer (ARET) strategy (**Figure 8J**). In the RAN-1 probe, MEHPPV, as an energy donor, can be triggered by AI producing^ 1^O_2_ to emit afterglow (AF1), and the AF1 energy was then transferred to the energy receptor (NRM) to release a longer wavelength afterglow (AF2) by the ARET effect. Upon treatment with NO, the weak electron acceptor (benzo[c]^1^,^2^,^5^ thiadiazole-5,6-diamine) of NRM reacted with NO to generate a stronger acceptor (5H[1,2,3]triazolo[4,5-f]-2,1,3-benzothiadiazole) of NRM-NO, which red-shifted its emission wavelengths due to enhanced effects of intramolecular charge transfer (ICT). As shown in** Figure 8K**, the FL intensity at 660 nm gradually increased and at 830 nm significantly increased with the increase of NO concentration, while the FL intensity of RAN-1 at 600 nm decreased. As shown in **Figure 8L**, after the incubated solutions were pre-irradiated by a 660 nm laser, a brighter AF2 at 830 nm and darker AF1 at 600 nm as a function of NO concentration was observed. Therefore, there was a good positive correlation between NO concentration and afterglow signal ratio (AF2/AF1) (**Figure 8M**). The RAN-2 was a ONOO^-^ responsive probe (**Figure 8N**). Upon treatment with ONOO^-^, as shown in** Figure 8O**, the FL intensity of the probe at 750-850 nm gradually decreased with the increase of ONOO^-^ concentration. Furthermore, the signal at AF1 (820 nm) became darker, while at AF2 (600 nm) remained almost unchanged with the increase of ONOO^-^ concentration (**Figure 8P**). Therefore, the AF2/AF1 signal ratio was linearly correlated with ONOO^-^ concentration. After intravenous administration, the AF2/AF1 ratio in inflammation model mice was higher than that in healthy mice (**Figure 8Q, R**), suggesting that RAN-1 could sense NO levels in inflammation model. These results indicated that RAN-1 and RAN-2 were excellent ratiometric afterglow probes for sensing of NO and ONOO^-^, respectively.

### Sensing of enzyme

**Figure 9:**
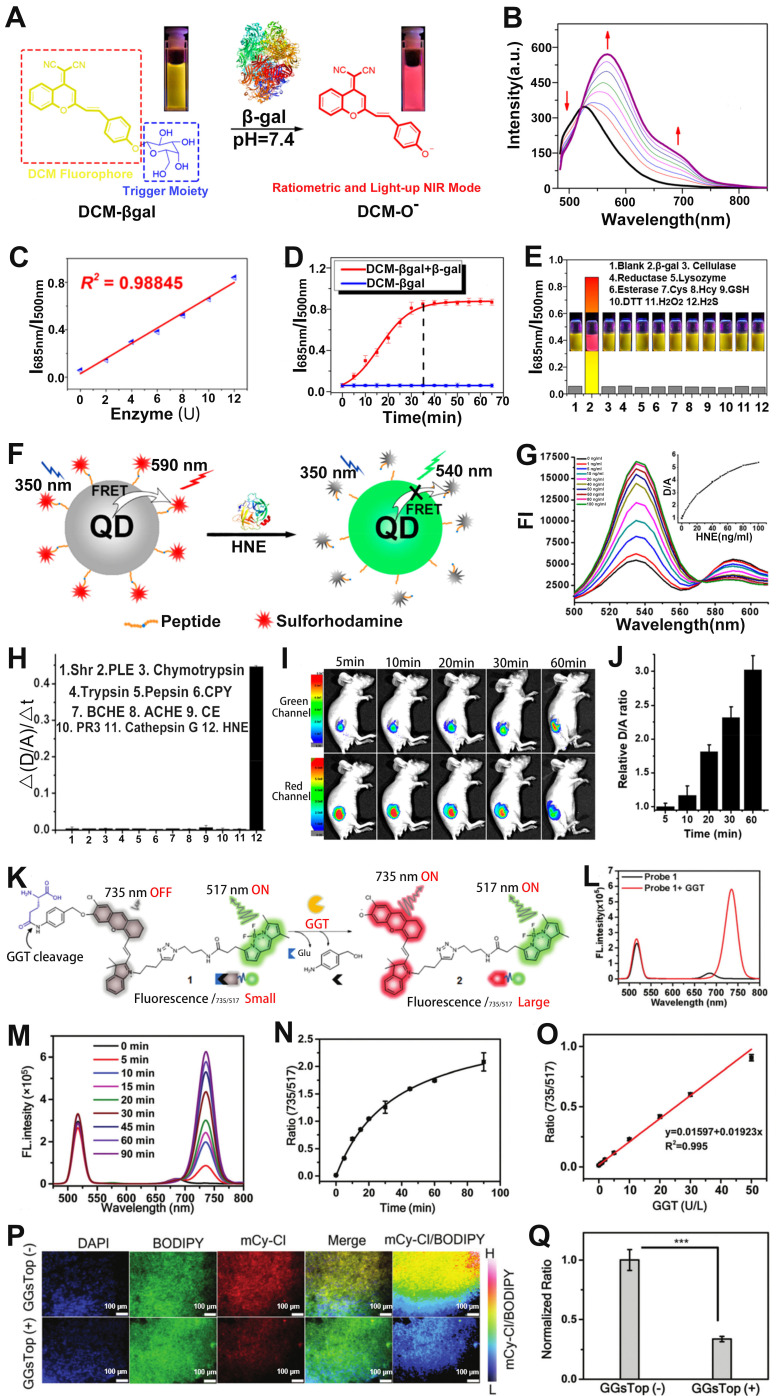
(**A**) Sensing mechanism of DCM-βgal under the action of *β*-gal enzyme. (**B**) ​Emission spectrum changes as a function of time. (**C**) Changes in FL signal ratio with increasing enzyme concentration. (**D**) FL signal ratio changes with time before and after addition of β-gal enzyme. (**E**) Changes in the FL ratio after treatment with different analytes. (**F**) Schematic diagram of sensing principle of QDP probe. (**G**) Changes in the FL spectra after treatment with different concentrations of HNE. (**H**) Changes in signal ratio after different analyte treatments. (**I**) FL images of tumor at different post-injection, and (**J**) corresponding D/A ratio. (**K**) The chemical structure of probe 1 and the principle of enzyme sensing. (**L**) FL spectra of probe after reaction with GGT. (**M**) ​FL spectra of the probe as a function of time after reaction with GGT. (**N**) Change of FL signal ratio with time. (**O**) The relationship between GGT concentration and FL signal ratio (FL_735_/FL_517_). (**P**) FL imaging of GGT activity in tumor tissue sections, and (**Q**) corresponding ratio (FL_735_/FL_517_). (**A-E**) Adapted with permission from 130, Copyright 2016 American Chemical Society. (**F-J**) Adapted with permission from 134, Copyright 2020 American Chemical Society. (**K-Q**) Adapted with permission from 135, Copyright 2021 Royal Society of Chemistry.

Enzymes participate in a wide range of important metabolic processes and are closely related to the activities of life 126-129. Real-time sensing of enzymatic activity is of great significance for cancer diagnosis. Zhu and co-workers reported a *β*-gal enzyme activatable ratiometric near-infrared (NIR) FL probe (DCM-βgal) consisting of dicyanomethyl-4H-pyran (DCM) chromophore (NIR FL reporter) and *β*-gal cleavable unit (enzyme-active trigger) for sensing of *β*-gal enzyme (**Figure 9A**) 130. DCM has the ability to regulate the electron donor of the phenolic group, and excellent donor-π-acceptor (D-π-A) characteristics. After DCM-βgal was incubated with *β*-gal enzyme, the C-O bond of DCM was hydrolyzed and released an electron-rich aglycon DCM-O^-^, which enhanced ICT effect and significantly changed its emission wavelength. As shown in **Figure 9B**, with the increase of *β*-gal concentration, the FL signal of the probe at 500 nm was weakened and a new peak appeared at 685 nm due to the increase of DCM-O^-^ of the pyrolysis product generated by the hydrolysis of DCM-βgal. Therefore, the ratio signal (I_685nm_/I_500nm_) increased linearly with the *β*-gal concentration in the range of 0-12 μM (**Figure 9C**). As shown in **Figure 9D**, the FL signal ratio was significantly increased after the addition of* β*-gal, and an approximately 14-fold enhancement in the FL ratio was observed after incubation for 35 min. Furthermore, the probe possessed good specificity for *β*-gal (**Figure 9E**), therefore, the ratiometric FL probe can be used to sense the activity of *β*-gal.

As a typical protease, human neutrophil elastase (HNE), is involved in pathogen destruction and regulation of inflammatory processes in the respiratory tract, and is associated with a variety of lung diseases 131. Therefore, designing ratiometric optical probes for *in vivo* sensing of HNE will provide a new approach for the clinical diagnosis of lung diseases. The quantum dots (QDs), characterized by high extinction coefficient, wide absorption spectrum and narrow emission spectrum, color tunability, long excited-state lifetime, *etc*., as FRET donors have been used for the sensing of disease-related biomarkers 132, 133. For example, Yang *et al.* developed a retiometric FL probe (QDP) consisting of CdSe/ZnS QDs as the FRET donor, sulforhodamine B (Rh) as the FRET acceptor and HNE-specific oligopeptide substrate (QPMAVVQSVPQK) for sensing of HNE (**Figure 9F**) 134. The emission spectra of the probe's donor (CdSe/ZnS QDs) overlap a large part of the absorption spectrum of its acceptor (Rh), forming a superior FRET effect. When the QDP probe was incubated with HNE, the amide bond between the two valines of the oligopeptide substrate was hydrolyzed by HNE, resulting in a disruption of the FRET effect, accompanied by a resumption of green FL from the donor and a decrease of red FL from the acceptor (**Figure 9G**). Therefore, the FL signal ratio (F_Donor_/F_Acceptor_, D/A) increased with increasing HNE concentration. As shown in F**igure 9H**, the QDP probe showed excellent specificity for HNE. When the QDP probe was injected *in situ* into the mouse of lung cancer model, the green FL gradually increased and the red FL gradually decreased at the tumor region as a function of time, leading to a time-dependent FL signal ratio (D/A) increased (**Figure 9I, J**). These results suggested that the FRET-based ratiometric FL probe possessed excellent sensitivity to HNE and, therefore, can be used to sense enzymes that were overexpressed in tumors.

Ye and colleagues developed a γ-glutamyl transpeptidase (GGT) responsive ratiometric FL probe with a FL emission at 517 nm for real-time ratiometric sensing of GGT. The ratiometric FL probe was comprised of GGT responsive cleavable amino acid substrate (γ-Glu), the self-immolation linker 4-aminobenzyl alcohol (PBAB), the quenched-near-infrared fluoresce-Cl, and the always-on BODIPY fluorescein (**Figure 9K**) 135. Upon incubation with GGT, the γ-Glu in probe was cleaved, subsequently triggered the spontaneous elimination of PBAB, affording a fluorescent product with a new FL emission at 735 nm (**Figure 9L**). As shown in **Figure 9M,** the FL intensity at 735 nm became stronger while at 517 nm remained almost unchanged with the increase of the reaction time between probe and GGT. Therefore, the FL signal ratio (I_735_/I_517_) increased as a function of time (**Figure 9N**), and exhibited a GGT concentration dependent I_735_/I_517_ ratio enhancement (**Figure 9O**). The ratiometric FL probe was incubated with Hela tumor sections and treated with GGT inhibitor (GGsTop) (**Figure 9P**), as shown in** Figure 9Q**, the FL signal ratio of the GGsTop treated group was significantly reduced compared with the GGsTop non-treated group, demonstrating that the probe had excellent GGT responsiveness and was a promising diagnostic tool for GGT sensing.

### Sensing of metal ions

**Figure 10:**
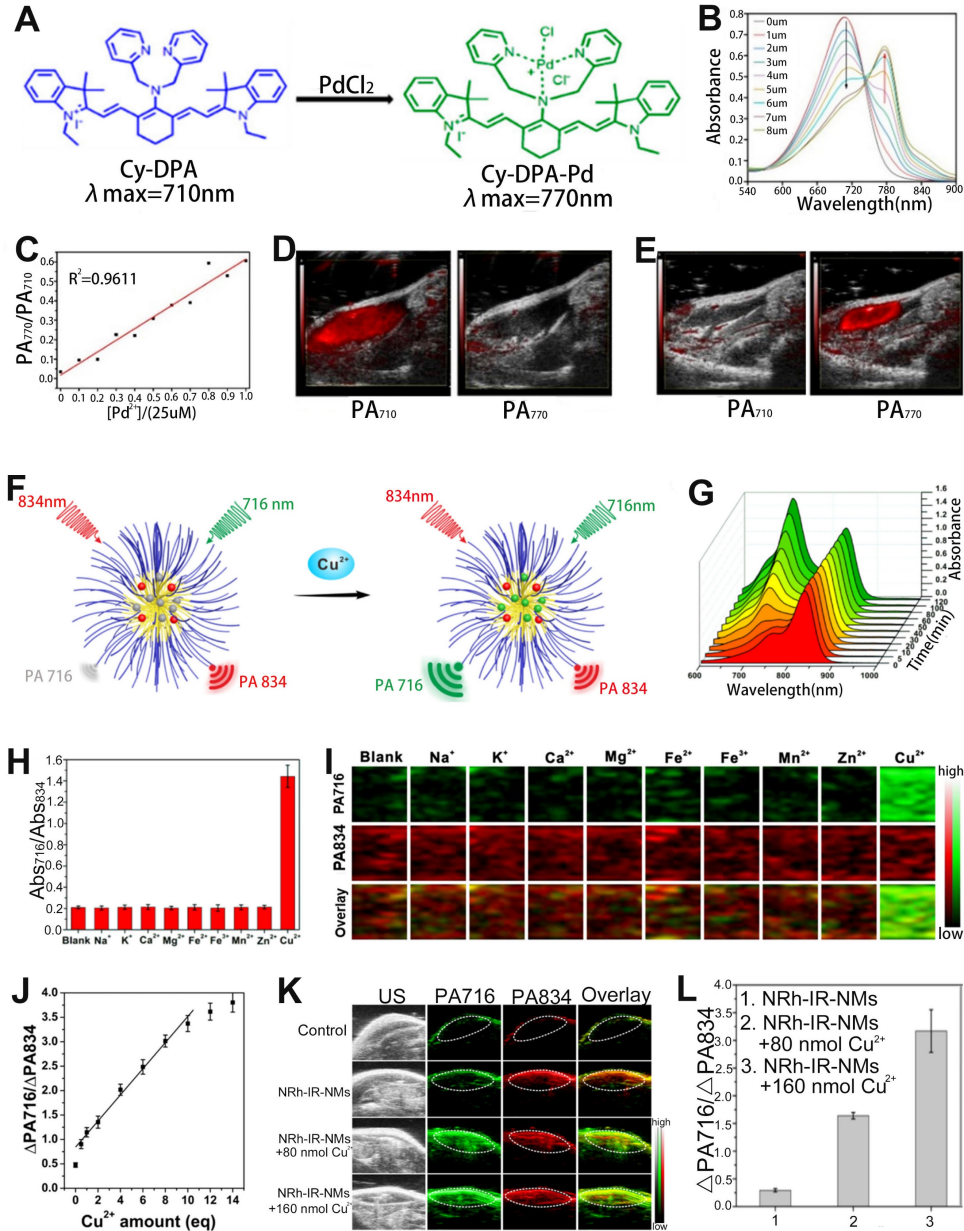
(**A**) Sensing mechanism of Cy-PDA for Pd^2+^. (**B**) The absorption spectrum of the Cy-PDA after adding different concentrations of Pd^2+^. (**C**) PA signal ratio (PA_770_/PA_710_) as a function of Pd^2+^ concentration. (**D**) PA images at 710 nm and 770 nm after injection of Cy-DPA through the groin in mice. (**E**) PA images at 710 nm and 770 nm after co-injection of Cy-DPA and Pd^2+^ through the groin in mice. (**F**) The sensing mechanism of NRh-IR-NMS for Cu^2+^. (**G**) Change of absorption spectra of NRh-IR-NM solution with time after adding Cu^2+^ solution. (**H**) Changes in Abs_716_/Abs_834_ of probes after incubation with different metal ions. (**I**) PA images of NRh-IR-NMS after reaction with metal ions. (**J**) The relationship between Cu^2+^ concentration and PA signal ratio. (**K**) PA images of mice after different treatments and (**L**) corresponding ΔPA_716_/ΔPA_834_. (**A-E**) Adapted with permission from 139, Copyright 2020 American Chemical Society. (**F-L**) Adapted with permission from 143, Copyright 2018 American Chemical Society.

Palladium (Pd) is an inert metal that enters the body by residing it in drugs such as gefitinib 136. When Pd residues enter the human body, excess palladium can lead to disruption of normal cellular functions, which in turn can cause multiple diseases. Recently, ratiometric optical probe has been investigated for sensing of metal ions 137, 138. For example, Cai *et al*. developed a cyanine fluorophore-based ratiometric PA Pd^2+^ sensor (Cy-DPA) for *in vivo* detection of Pd^2+^ 139. The ratiometric PA sensor was composed of a heptamethine cyanine as the reporter unit and a dimethylpyridinamine as the recognition unit (**Figure 10A**). As shown in **Figure 10B**, the absorbance of the sensor at 710 nm gradually decreased, while at 770 nm gradually increased with the increase of Pd^2+^ concentration, due to the change of electronic structure after the interaction between Cy-PDA and Pd^2+^. Therefore, the PA intensity ratio (PA_770_/PA_710_) increased with increasing Pd^2+^ concentration (**Figure 10C**). After groin injection of Cy-DPA, the injection region of mice showed a high PA signal at 710 nm and a negligible signal at 770 nm (**Figure 10D**). However, upon Pd^2+^ in solution was injected into the same location, as shown in **Figure 10E**, the signal at 770 nm increased, while at 710 nm decreased. Therefore, the ratiometric PA sensor (Cy-DPA) was effective for* in situ* sensing of Pd^2+^.

Divalent copper ion (Cu^2+^) is an essential metal element for the human body, but abnormal copper content can cause numerous serious diseases 140-142. Therefore, the development of reliable Cu^2+^ sensing methods is of great significance for the diagnosis and treatment of diseases. Chen and colleagues developed a ratiometric PA probe (NRh-IR-NMS) consisting of a selective Cu^2+^ response NRh (as a sensing signal), Cu^2+^ insensitive dye IR (as an internal reference signal) with a PA signal at 834 nm and nanomicelles (NMS) for the sensing of Cu^2+^ *in vivo* (**Figure 10F**) 143. ​The NRh-IR-NMS reaction with Cu^2+^ opened the spirolactam ring to generate NRh1 upon treatment with Cu^2+^, resulting in a significant enhancement of the absorbance at 716 nm (**Figure 10G**). In addition, the NRh-IR-NMS probe showed high selectivity for Cu^2+^ (**Figure 10H**, **I**), and the PA signal ratio (PA_716_/PA_834_) increased linearly with the increase of Cu^2+^ concentration (**Figure 10J**). After NRh-IR-NMS probe and Cu^2+^ were co-injected subcutaneously into mice, the PA images at injection region exhibited a Cu^2+^ concentration dependent PA signal ratio (PA_716_/PA_834_) enhancement (**Figure 10K**, **L**). These results illustrated that the ratiometric PA probe was an excellent sensing platform for disease diagnosis due to its high selectivity and deep tissue penetration capability.

In addition to fluorescent dyes, metal-organic frames (MOFs) prepared by metal ions (or metal clusters) and organic systems with the high sensitivity of luminescence detection and sensing accuracy 144-147 can also realize ratiometric FL sensing of metal ions such as Cu^2+^ and Fe^3+^ by using the ratio of the two-emission intensity 148, 149.

### Sensing of GSH

**Figure 11:**
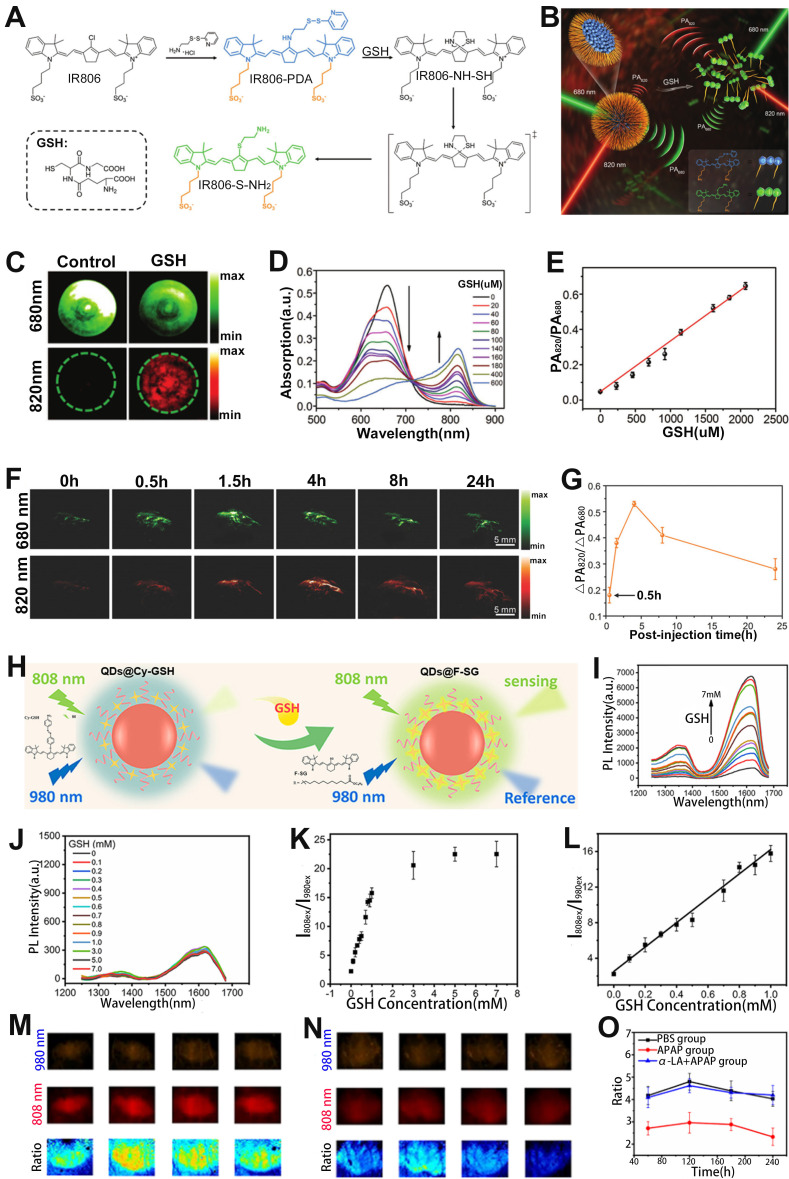
(**A**) Molecular structure of the IR806-PDA probe and the reaction pathway with GSH. (**B**) Proposed mechanism of the ratiometric probe for GSH sensing. (**C**) PA imaging at 680 nm and 820 nm of probes after different treatments. (**D**) GSH responsive UV-vis absorption spectra of IR806-PDA nanoprobe. (**E**) Relationship between GSH concentration and PA signal ratio. (**F**) PA images at different post-injection time. (**G**) Relationship between ΔPA_820_/ΔPA_680_ and post-injection time. (**H**) Schematic illustration of the ratiometric FL probe for sensing of GSH. FL spectra of probe incubated with different concentrations of GSH under excitation at (**I**) 808 nm and (**J**) 980 nm. (**K**) The relationship between FL signal ratio and GSH concentration. (**L**) GSH concentration as a function of FL signal ratio. FL signal intensity in mouse liver treated with (**M**) PBS and (**N**) APAP. (**O**) The change of FL signal ratio (I_808ex_/I_980ex_) with time after different treatments. (**A-G**) Adapted with permission from 152, Copyright 2018 WILEY-VCH. (**H-O**) Adapted with permission from 153, Copyright 2021 American Chemical Society.

GSH is involved in the etiology and development of numerous diseases 150, 151. Therefore, GSH sensing is essential for better disease treatment and understanding of pathological phenomena. Fan and colleagues designed a cyanine derivative-based activatable probe IR806-pyridine dithioethylamine (PDA) consisting of PDA and a disulfide bridged IR806 for real-time sensing of GSH *in vivo* (**Figure 11A, B**) 152. After incubation with GSH, the disulfide bond of the probe was broken and reduced to sulfydryl group (-SH) due to the extrusion of pyridine, and the product IR806-NH-SH was generated, resulting in wavelength red-shift from 680 nm to 820 nm. Therefore, the PA signal of the probe at 680 nm gradually decreased, while at 820 nm increased with the increase of GSH concentration, resulting in a GSH concentration dependent enhanced PA signal ratio (PA_820_/PA_680_) (Figure 11C-E). In addition, the PA signal ratio of GSH treated probe was 12.77-fold and 13.09-fold higher than that of cysteine- and homocysteine-treated probe, respectively. After intravenous injection, ΔPA_680_ increased slightly over time and reached the maximum at 1.5 h post-injection due to the synergistic effect of probe activation leading to a weakened PA signal at 680 nm and probe accumulation in the tumor region, while ΔPA_820_ exhibited a significant increased and reached the plateau at 4 h post-injection (**Figure 11F**). Thus, the PA ratiometric intensity of IR806-PDA in a HeLa tumor model remarkably increased at 4 h post-injection (**Figure 11G**). These results demonstrated that the ratiometric PA probes can be used to specifically sense and identify GSH.

In addition to the ratiometric PA probe, the ratiometric FL probe can also be used to sense GSH. For example, Liu *et al.* developed an activatable ratiometric FL probe (QDs@Cy-GSH) consisting of an organic dyes Cy-GSH, DSPE-PEG and Ag_2_Te QDs for sensing of GSH (**Figure 11H**) 153. The QDs@Cy-GSH probe emitted faint light at 1620 nm under the excitation of 808 nm due to the PET effect from cyanine to the nitroazo group. After the probe was incubated with GSH, GSH replaced the nitro in the probe to form F-SG products, resulting in the elimination of PET effect, hence the FL signal at 1620 nm under 808 nm laser excitation was enhanced, while the FL signal under 980 nm excitation remained unchanged (**Figure 11I, J**). Therefore, the FL signal ratio (I_808ex_/I_980ex_) increased as a function of GSH concentration (**Figure 11K, L**). After intravenous administration, the probes were mainly accumulated in the liver because of the overexpression of GSH in the liver. Excess GSH in liver was produced through APAP induced liver injury. After treatment, a stronger I_808ex_/I_980ex_ in APAP group and weaker I_808ex_/I_980ex_ in APAP+α-lipoic acid (α-LA) group was observed (**Figure 11M-O**) due to the α-LA inhibited liver toxicity. Therefore, the GSH level could be sensed through the variation of ratiometric FL signal (I_808ex_/I_980ex_).

### Sensing of Gas molecules

**Figure 12:**
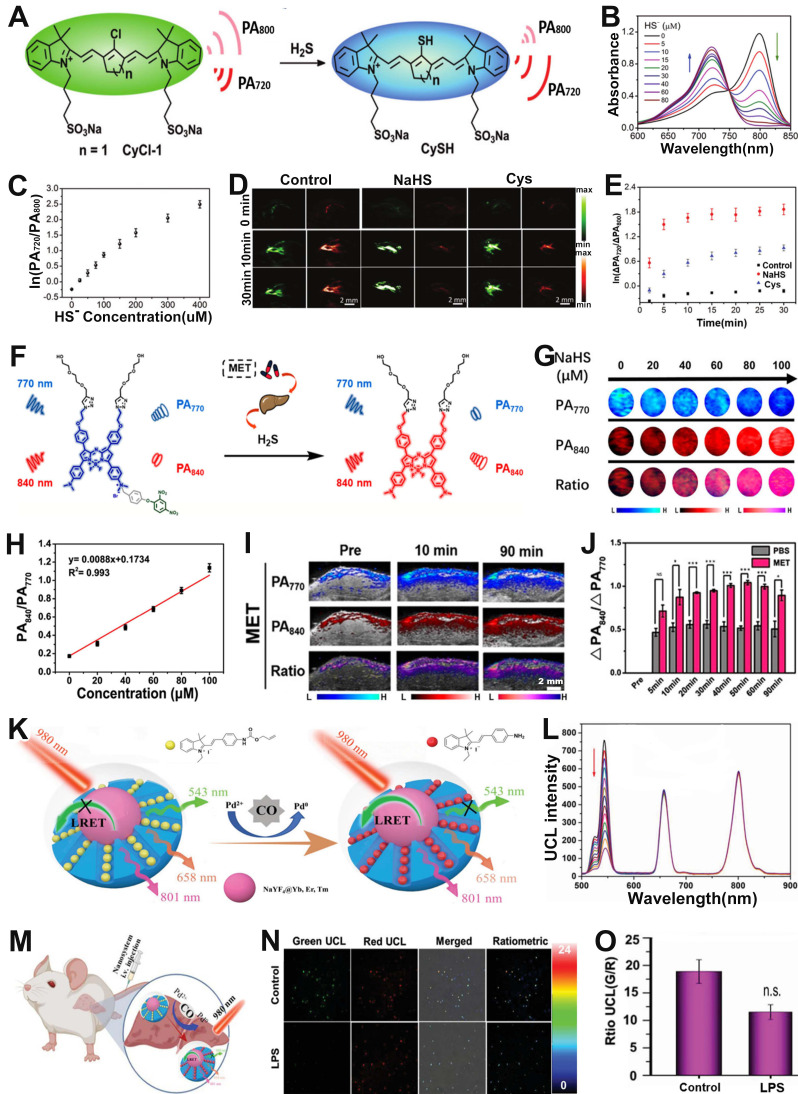
(**A**) Schematic illustration of the structure and sensing principle of CyCl-1 probe. (**B**) Changes in the absorption spectra of probes with increasing HS^-^ concentration. (**C**) The ln(PA_720_/PA_800_) as function of HS^-^ concentration. (**D**) PA images of mice after different treatments. (**E**) The time-dependent ΔPA signal ratio change. (**F**) Schematic diagram of the sensing principle of BDP-H_2_S probe. (**G**) PA images after treatment with various NaHS concentration, and corresponding (**H**) PA_840_/PA_770_. (**I**) PA images of the liver in mice with MET-induced liver injury. (**J**) PA_840_/PA_770_ in PBS group and MET-induced liver injury group. (**K**) Schematic illustration of the ratiometric UCL probe for CO sensing. (**L**) The absorption spectrum of the ratiometric probe after adding different concentrations of CO. (**M**) Schematic illustration of CO sensing in liver tissues of mouse. (**N**) Ratiometric imaging of endogenous CO in mouse liver tissue after different treatments and (**O**) corresponding UCL_534 nm_/UCL_658 nm_ ratio. (**A-E**) Adapted with permission from 157, Copyright 2019 Royal Society of Chemistry. (**F-J**) Adapted with permission from 158, Copyright 2022 American Chemical Society. (**K-O**) Adapted with permission from 166, Copyright 2022 Wiley-VCH.

Hydrogen sulfide (H_2_S), NO and carbon monoxide (CO) are three endogenous gaseous transmitters that are essential regulators of a variety of physiological and pathological processes 154-156. ​Abnormal production of H_2_S can cause a variety of diseases. Therefore, designing ratiometric probes to quantitatively sense H_2_S *in vivo* is essential for better disease treatment and prognosis. Fan and colleagues developed a chlorinated cyanine dyes based ratiometric PA probe (CyCl-1) for sensing of H_2_S *in vivo* through nucleophilic substitution reactions of H_2_S with chlorine (**Figure 12A**) 157. Upon treatment with H_2_S, chlorine in the probe was replaced by HS^-^, resulting in a change in the color of the probe solution from green to blue due to the ICT effect. In addition, the absorption intensity at 800 nm reduced while that at 720 nm enhanced with the increase of HS^-^ concentration (**Figure 12B**), resulting in a HS^-^ concentration dependent PA_720_/PA_800_ enhancement (**Figure 12C**). After injection of probe into PBS, NaSH and Cys (a precursor of H_2_S) treated mice, respectively, the PA signal ratio in NaHS and Cys treated group increased, while that in PBS treated group remained almost unchanged (**Figure 12D, E**), indicating the CyCl-1 probe had excellent responsiveness to H_2_S and the correlation between H_2_S concentration and PA_720_/PA_800_ signal ratio enabled quantitative evaluation of endogenous H_2_S.

In addition, ratiometric optical probe can be used to sense H_2_S in drug-induced liver injury. For example, Song *et al.* developed a H_2_S activatable ratiometric PA probe (BDP-H_2_S) consisting of aza-BODIPY dye as the signaling moiety and 2,4-dinitrophenyl (DNP) ether as the H_2_S responsive moiety for sensing of H_2_S in drug-induced liver injury (**Figure 12F**) 158. ​The DNP ether, which acted as a strongly electron withdrawing H_2_S-responsive moiety, was connected to the Aza-BODIPY scaffold, leading to a blocking of the ICT effect. Upon treatment with H_2_S, the ether bond was attacked and broken, and the quaternary ammonium of Aza-BODIPY was converted to tertiary amine. Therefore, the blocked ICT effect was recovered, causing a red-shift of the BDP-H_2_S absorption from 770 nm to 840 nm.

After the addition of NaHS, the absorption at 770 nm of the probes decreased and at 840 nm increased with increasing NaHS concentration, indicating the BDP-H_2_S probe had excellent responsiveness to HS^-^. Moreover, the same trend of change occurred for the PA signals (**Figure 12G**). Consequently, the PA signal ratio (PA_840_/PA_770_) increased linearly with HS^-^ concentration (**Figure 12H**). *In vivo* PA imaging in healthy BALB/c mice model and metformin (MET) induced liver injury model (**Figure 12I**) was used to sense H_2_S content. As shown in **Figure 12J**, the PA signal ratio (PA_840_/PA_770_) of PBS-treated mice was much weaker than that of MET treated mice at 90 min post-injection. The PA_840_/PA_770_ ratio in the MET-treated group reached a maximum at 50 min post-injection, and the intensity of the ratio was enhanced approximately 2-fold compared to the PBS group, indicating that the BDP-H_2_S probe enabled accurate sensing of *in vivo* H_2_S level in drug-induced liver injury and had promising application prospects in specific disease-related analytes sensing.

CO, as the second gasotransmitter, is involved in various physiological and pathological processes 159, 160. For example, it plays a very important role in neurotransmitter regulation, vasodilatation and anti-inflammatory effects 161-163. However, excessive CO can lead to heart failure (HF), hypertension and many other diseases 164, 165. It is therefore extremely valuable to develop effective tools to examine* in vivo* CO levels variations with high selectivity and sensitivity. Zhou and co-workers reported a luminescent resonance energy transfer (LRET) effect based ratiometric probe consisting of mesoporous silica (mSiO_2_)-coated UCNPs (NaYF_4_@Yb, Er, Tm) with absorption at 543 nm, 658 nm, 801 nm, CO-responsive hemicyanine derivation dye (CyD1) with absorption at 432 nm, and PdCl_2_ as an additive, for CO sensing *via* upconversion luminescent (UCL) imaging (**Figure 12K**) 166. CO reduced Pd^2+^ to generate Pd^0^ and subsequently mediated the Tsuji-Trost reaction to remove the allylcarbonate group of CyD1, leading to release of CyNH, therefore, the absorption peak was significantly redshifted from 432 nm (CyD1) to 522 nm (CyNH). The increased overlap of the absorption band of CyNH with the emission band of NaYF4@Yb, Er, Tm caused the quenching of the emission at 534 nm (green light) of NaYF4@Yb, Er, Tm, while the emission at 658 nm (red light) remained unchanged as an internal standard (**Figure 12L**). Therefore, the UCL_543_/UCL_658_ can be used as an output signal for accurate and quantitative sensing of CO. It has been shown that LPS can stimulate the production of endogenous CO. ​Mice were given saline or LPS intravenously after 12 h, followed by intravenous administration of the probe (**Figure 12M**). A significant decrease in the UCL_534 nm_/UCL_658 nm_ was observed only in the liver of LPS-treated mice, but not in saline-treated controls, due to LPS-induced CO production in the liver region (**Figure 12N, O**). In addition, the UCL_534 nm_/UCL_658 nm_ was reduced approximately 1.64-fold in the LPS-treated mice group compared to the saline-treated mice group. These results demonstrated that the ratiometric probe can be used for sensing of endogenous CO and provide an effective strategy for detecting disease-related gas molecules.

### Sensing of hypoxia

**Figure 13:**
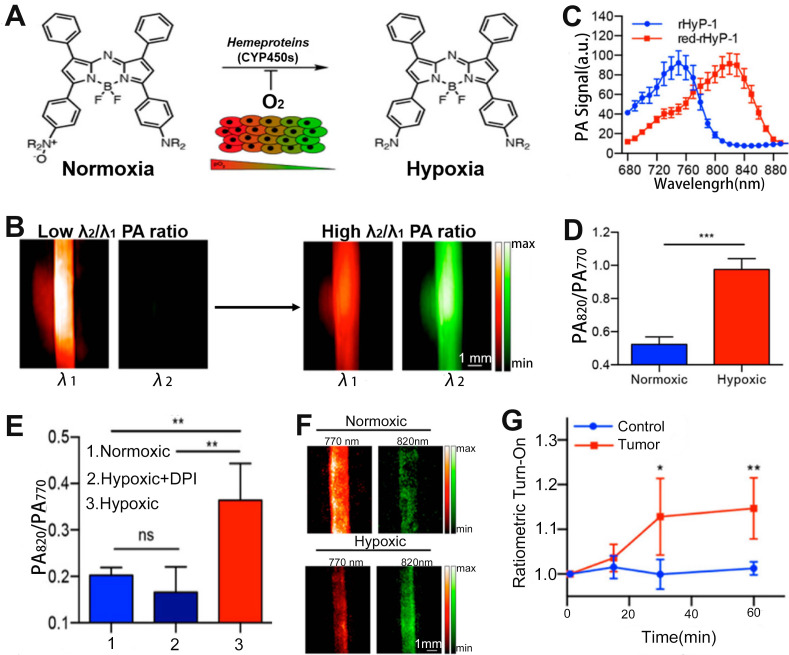
(**A**) Study on the sensing mechanism of probes for hypoxia. (**B**) Changes in PA signal ratio before and after hypoxia. (**C**) PA spectra of rHyP-1 and red rHyP-1. (**D**) Changes in PA signal ratio after 1 h incubation in hypoxic or normoxic conditions. (**E**) Changes of PA signal ratio after different treatment. (**F**) Representative PA images of rHyP-1 probe solutions after incubatiom with rat liver microsomes. (**G**) After rHyP-1 was injected intratumorally or subcutaneously (control) into the tumor-bearing mice, the ratiometric PA turn-on. (**A-G**) Adapted with permission from 171, Copyright 2018 American Chemical Society.

​Hypoxia is a pathogenic feature of solid tumors due to the absence or abnormal vasculature in the TME, and is essential in tumor progression, angiogenesis, metastasis, invasion, and resistance to immune systems and therapies 167-170. Therefore, it is of great importance to develop a ratiometric probe for the sensing and quantification of intratumoral hypoxia. Chan et al. designed a hypoxia-responsive ratiometric PA probe (rHyP-1), with a high and low PA signal ratio for hypoxia and normoxia, respectively, for reliable hypoxia sensing (Figure 13A, B) 171. In vitro ratiometric PA experiments on rHyP-1 indicated that the PA820nm/PA770nm ratio in hypoxia was twice that in normal conditions (Figure 13C-F). Furthermore, the rHyP-1 probe was able to not only sense the intratumor hypoxia, but also predict the region of the tumor where the probe was activated. As shown in Figure 13G, rHyP-1 was used in the sensing of hypoxia in a mouse model of a bearing tumor, where a clear enhancement of PA signal ratios was observed at the injection site, and the PA820nm/PA770nm ratiometric turn-on response relative to the control group increased by a factor of 1.2. In addition, the hypoxic region of tumor can be located via three-dimensional reconstruction ratiometric PA imaging due to the specific activation of the rHyP-1 probe by hypoxia. These results indicated that the ratiometric PA probe can be used to sense and localize the hypoxia in tumor regions.

### FRET-based ratiometric probe for immunoassay biosensing

​ FRET immunoassay is a type of biochemical test that is based on antibody-antigen interactions and allows for the direct and rapid detection of antibody-antigen complex assembly in solution, making it possible to quantify any target of interest in various types of clinical specimens 172, 173. To perform a FRET immunoassay, two different antibodies are used - one bound to a lanthanide-based donor and the other bound to an appropriate fluorescent receptor - that separately bind to different epitopes of a target antigen. This brings them into close proximity, enabling FRET. Thus, FRET immunoassay is a powerful tool in molecular diagnostics, with several commercially available brand names, such as HTRF or TRACE, being used clinically to detect different biomarkers 174. Moreover, time-resolved (TR) or time-gated (TG) FRET immunoassay, which has been used in clinical diagnosis for many years, is particularly useful in its ability to measure fluctuations in FL over time. By combining this with a sensitive detection method, FRET immunoassays can provide highly accurate and reliable diagnostic results for a range of clinical applications 175-177.

B-type natriuretic peptide (BNP) is a peptide hormone secreted by ventricular cells in response to an increase in ventricular wall tension 178. Patients with HF have a significant increase in BNP in the blood due to intravascular volume expansion and cardiac pressure overload 179. Therefore, BNP is considered to be a biomarker of HF. A highly sensitive and specific test for BNP is important for the diagnosis and prognosis of HF patients. Zhang *et al*. developed a FRET-based ratiometric probe for the sensing of BNP based on the competitive binding of graphene oxide (GO) and BNP to its aptamer 180. The FRET-based ratiometric probe was comprised of GO as the acceptor and a carboxyfluorescein-modified aptamer (FAM-aptamer) with specific recognition of BNP as the donor. GO can bind to FAM-aptamer through hydrophobic interaction and π-π stacking interaction, causing FL signal of FAM-aptamer to be quenched through FRET effect between GO and FAM-aptamer. ​However, in the presence of the BNP, the BNP preferentially bound to FAM-aptamer and induced a change in the aptamer structure, leading to a separation of GO and FAM-aptamer, after which the FRET effect disappeared and the FL signal of FAM-aptamer was thus restored. Therefore, BNP levels* in vivo* can be sensed by the FL signal ratio (F_donor_/F_acceptor_). The results indicated that the FRET-based ratiometric probe had good selectivity for BNP and can therefore be used for immunoassay biosensing.

## Conclusion and outlook

​The ratiometric optical probes and their applications in biosensing have attracted increasing attention over the past decade. Compared to traditional sensing methods for specific disease-related analytes, the ratiometric method enables accurate, real-time sensing with high sensitivity, deep tissue penetration, and self-calibration signal correction. This review systematically summarized and discussed the design strategies and mechanisms of ratiometric optical probes and their applications in biosensing.

​Although current research on ratiometric optical probes is developing rapidly and has made great progress in biosensing, there are endless possibilities for the development of new ratiometric optical probes that meet the sensing needs of different analytes. Accordingly, four research directions deserve our special attention:

(1) ​Since the same analyte may be present in the pathological microenvironment of different diseases, it is difficult to accurately diagnose a specific disease with a single disease-associated analyte activated ratiometric probe. Therefore, to improve the accuracy and specificity of disease sensing, an effective approach is to design a ratiometric probe that can be used for the sensing of two or multiple biomarkers of a specific disease.

(2) Although ratiometric sensing strategies with a single imaging modality effectively mitigate the problem of the interference of probe's concentration dependent factor, challenges remain due to the limitation of single model imaging. Multimodal ratiometric molecular imaging, such as a single probe with capability of ratiometric PA imaging and ratiometric FL imaging, will be an alternative option for precision medicine in the future. By integrating the advantages of two or multimodal imaging modalities into a single ratiometric probe can minimize the limitations of a single imaging modality and provide more comprehensive information, which is of great value in the diagnosis and treatment of diseases.

(3) Designing a single probe with the ratio between two different imaging modalities could also be a future direction. For example, CL is a self-luminescent imaging method that can avoid the limitations of FL in the presence of strong tissue autofluorescence and light scattering, while FL imaging has high sensitivity and specificity, thus ratiometric CL/FL probe can realize complementary advantages.

(4) Biological barriers in the transport of ratiometric probes hinder biosensing of specific organelles in living cells or target tissues* in vivo*. This process severely limits the bioavailability of the probe at a specific size, which in turn compromises excellent sensing outcomes. The non-specific distribution and inadequate delivery of ratiometric probes are current issues that still need to be addressed. In the next study, ratiometric probes can be rationally combined with innovative targeting design features to overcome biological barriers and realize better sensing performance.

​In conclusion, with increasing progress and continuous innovation in materials science and sensing technologies, it is reasonable to believe that ratiometric optical probes are expected to have a broad future in biomedical field.

## Figures and Tables

**Table 1:** Summary of the merits and drawbacks of different optical modalities

Classification	Merits	Drawbacks	Ref.
PA imaging	Deep tissue penetration; high imaging resolution, low scattering and dissipation in biological tissue	Low signal-to-noise ratio; diminished image contrast due to strong optical attenuation; a lower-bound on spatial resolution in deep tissue	24-26
FL imaging	Excellent sensitivity and selectivity; high spatiotemporal resolution; real-time detection; non-invasiveness; and low cost	Limited tissue penetration depth; severe interference from tissue absorption, scattering, and spontaneous fluorescence	27-29
BL imaging	No autofluorescence and phototoxicity; without external light excitation	Bioluminescence signal is relatively low and bioluminescence imaging rely on enzyme-initiated redox reactions to trigger luminescence	30-32
CL imaging	Effectively avoids light scattering; high sensitivity and signal-to-noise ratio, without external light excitation, no phototoxicity	Chemiluminescence signal is relatively weak and chemiluminescence signals are easily perturbed by internal stimuli such as redox microenvironment	33-36
Afterglow luminescence imaging	Effectively eliminated autofluorescence; no particular chemical mediator or exogenous enzyme	Luminescence decays with time, poor quantitative ability	37-39

## Abbreviations

ACIE: absorption competition induced emission
AI: afterglow initiator
α-LA: α-lipoic acid
ARET: afterglow resonance energy transfer
BG: bioactive Glass
BL: bioluminescence
BNP: B-type natriuretic peptide
CFP: cyan fluorescent protein
CL: chemiluminescence
CO: carbon monoxide
CRET: chemiluminescence resonance energy transfer
Cu^2+^: divalent copper ion
CyD1: hemicyanine derivation dye
D-π-A: donor-π-acceptor
DCM: dicyanomethyl-4H-pyran
DCNP: down-conversion nanoparticles
DNP: 2,4-dinitrophenyl
DSPE-PEG: polyethylene glycol phospholipids
ErNPs: Er nanoparticles
EYFP: enhanced yellow fluorescent protein
FAM-aptamer: carboxyfluorescein-modified aptamer
FL: fluorescence
FRET: fluorescence resonance energy transfer
GGsTop: GGT inhibitor
GGT: γ-glutamyl transpeptidase
GM-Ag_2_S NDs: co-modified Ag_2_S nanodots
GO: graphene oxide
GSH: glutathione
HF: heart failure
H_2_S: hydrogen sulfide
HNE: human neutrophil elastase
H_2_O_2_: hydrogen peroxide
HOCl/HClO: hypochlorous acid
ICT: intramolecular charge transfer
IR-NO: NO-responsive small-molecule organic dye
LPS: lipopolysaccharide
LRET: luminescent resonance energy transfer
MEHPPV: afterglow substrate
MET: metformin
minocycline: OONO^-^ scavenger
MPA: mercaptopropionic acid
MOFs: metal-organic frames
MPS/mSiO_2_: mesoporous silica
mTurquoise2: CFP variant
NIR: near-infrared
NMS: nanomicelles
NO: nitric oxide
NO_2_^•^: nitrogen dioxide radicals
NO_3_^-^: nitrate
NRM: NO-responsive molecule
O_2_^·-^: superoxide
^1^O_2_: singlet state oxygen
^-^OCl: hypochlorite
·OH: hydrogen radicals
ONOO^-^: peroxynitrite
PA: photoacoustic
PBAB: 4-aminobenzyl alcohol
Pd: Palladium
PDA: pyridine dithioethylamine
PEG-b-PPG-b-PEG: amphiphilic triblock copolymer
PET: photoinduced electron transfer
pH-BDP: pH indicator and enhancer
QDs: quantum dots
RAN: ratiometric afterglow probes
Rh: sulforhodamine B
RNS: reactive nitrogen species
ROS: reactive oxygen species
SeTT: NIR-II FL dye
SIN-1: OONO^-^ donor
SO: semiconducting oligomer
SOA: semiconducting oligomer amphiphile
-SH: sulfydryl group
TG: time-gated
TME: tumor microenvironment
TR: time-resolved
UCL: upconversion luminescent
UCNPs: upconversion nanoparticles
γ-Glu: GGT responsive cleavable amino acid substrate
